# Announcement: Vth COMTOX Symposium on Toxicology and Clinical Chemistry of Metals

**DOI:** 10.1155/MBD.1994.343

**Published:** 1994


					Vth COMTOX SYMPOSIUM
ON TOXICOLOGY AND CLINICAL
CHEMISTRY OF METALS
Vancouver, British Columbia, Canada
10 to 13 July 1995
Site and date
The Vth COMTOX Symposium will be held at the University of British Columbia on 10 to
13 July 1995, in the week between the VIIth International Congress of Toxicology in
Seattle, Washington, and the American Association for Clinical Chemistry meeting in
Anaheim, California. The Symposium is a Satellite Meeting of the International Congress of
Toxicology.
The Four Major Themes of the Scientific Program
Analysis of Metals in Biological Materials
Inductively coupled plasma mass spectrometry and atomic emission spectroscopy;
electrothermal atomic absorption spectrometry; neutron activation analysis and related
techniques, electron microprobe analysis; voltametry and related techniques; speciation
by high performance liquid chromatography; capillary electrophoresis with metal-
sensitive detectors; metal receptor isolation by metal-affinity chromatography; Western
blots with metal radioprobes; reference methods and materials; quality assurance, and
interlaboratory harmonization of metal analyses.
Molecular Biology & Toxicology of Metals
Cellular entry and subcellular translocation of metals; metal-binding to macromolecules
and diffusible ligands; metallothionein and its isoforms; metals in cell division, gene
replication, and transcription; metal-induced free reactions; mechanisms of metal
genotoxicity, carcinogenesis, and teratogenesis.
Metals in Health and Disease
Metal absorption, distribution, and kinetics; animal models; nutritional essentiality;
dietary requirements; reference values for metals in body fluids and tissues; toxicity for
brain, liver, kidney, heart, lung, bone, and skin; hematological, immunological,
endocrine, and reproductive effects; interactions of essential and toxic trace metals;
genetic disorders of metal metabolism; inter-individual variation in susceptibility to
metal toxicity; metal accumulation in organ insufficiency; metal compounds as drugs and
chemotherapeutic agents; chelation treatment and dietary therapy for metal overload.
Occupational & Environmental Exposures
Chemical and biological monitoring of metal exposures from occupational, environmental,
and iatrogenic sources; metal release from dental and orthopaedic prostheses; biomarkers
of metal toxicity and carcinogenesis; epidemiological studies.
343
Vol. 1, No. 4, 1994 Announcements
Lectures
Trace Metal Analysis
Chemical speciation of metals in biological materials. R Cornelis (Belgium)
Trace metal isotopes in body fluids. D Templeton (Canada)
The impact of laboratory design on analytical sensitivity for trace metals. TP Moyer
(USA)
Quality control of trace element measurements in body fluids and tissues. Y Thomassen
(Norway)
uality assurance of blood lead assays for screening and diagnostic purposes. PJ Parsons
(USA)
Analytical imaging of chemical elements and their isotopes in biological materials. ME
Thellier (France)
Sources of error in ICP-MS analyses of trace metals in biological materials. NI Ward
(UK)
Metals and Gene Regulation
Metal interactions with DNA-binding proteins. B Sarkar (Canada)
Metalloregulatory transcription factors in eukaryotic gene expression. DJ Thiele (USA)
Regulation of the mouse metallothionein gene expression. C Sdguin (Canada)
Genetic alterations of glutathione and porphyrin synthesis as adaptive responses to
mercury-induced oxidative stress. JS Woods (USA)
Interference of cadmium with calcium-mediated gene expression. D Beyersmann
(Germany)
Regulation of gene expression and hormone signal transduction by lead in osteocytes. J.
Pounds (USA)
Regulation and Disorders of Trace Metal Metabolism
Molecular analysis of genetic disorders of copper metabolism in humans and animals. JB
Mercer (Australia)
Control of metallothionein degradation. Bremner (UK)
Copper mobilization following ischemic myocardial injury. M Chevion (Israel)
Pathological consequences of disordered copper metabolism in LEC rats. N. Takeichi
(Japan)
Hereditary hepatitis caused by disordered metabolism of copper in LEC rats; recovery by
chelation therapy. KT Suzuki (Japan)
Metal Compounds as Drugs
Metal compounds in cancer chemotherapy. N Imura (Japan)
Clinical pharmacokinetics and therapeutic applications of bismuth compounds. KM Koch
(USA)
Metal Carcinogenesis
Role of oxidative damage in metal-induced carcinogenesis: Recent developments. KS
Kasprzak (USA)
Mutations induced by metal generated oxygen reactive species. L. Loeb (USA)
Interaction of carcinogenic metal compounds with DNA repair processes. A Hartwig
(Germany)
Genotoxicity and carcinogenicity of chromium compounds: Mechanistic aspects. S. DeFIora
(Italy)
Nickel carcinogenesis: Inherited effects on transcription. M. Costa (USA)
Cytoskeletal injury induced by carcinogenic metals. IN Chou (USA)
Anti-carcinogenic activity of cadmium. M. Waalkes (USA)
344
Metals and Nutrition
Recent advances in knowledge about the essentiality of trace metals. M. Anke (Germany)
Chronic studies of the absorption and metabolism of selenium by animals and humans. P.
Whanger (USA)
Selenium nutrition in pediatrics. G. Lockitch (Canada)
Paper on zinc nutrition and metabolism. (to be announced)
Recent advances in iron nutrition and metabolism. S. Hercberg (France)
Manifestations of chromium deficiency in human and animal nutrition. RA Anderson (USA)
Metals and Reproduction
Molecular targets for metal teratogenesis. FW Sunderman Jr. (USA)
Some molecular effects of metals on reproduction and development of the embryo and fetus.
NK Mottet and R. Ponc (USA)
Mathematical modeling for risk assessment of the teratogenicity of methylmercury. E.
Faustman (USA)
Toxicity of Metals
Molecular and cellular approaches to understanding mechenism of metal toxicity. MG
Cherian (Canada)
Mechanisms of III-V semiconductor toxicity. B. Fowler (USA)
Factors influencing the bioavailability of dietary lead and cadmium. M. Vahter (Sweden)
Osteotoxicity of metal compounds: Relevance to human exposures. M. Bhattacharyya (USA)
The immunotoxicity of metals. R. Smialowicz (USA)
Metallothionein and the immune system. AP Autor (Canada)
Prevention of nickel dermatitis by regulation of specific exposures. T. Menn (Denmark)
Occupational and Environmental Exposures to Metals
Role of metals in neurodegeneration. J. Savory (USA)
Defining the critical organs and adverse effects of metals in humans. G. Nordberg
(Sweden)
Multiple exposures to metals in the workplace. L. Alessio (Italy)
Clinical data for occupational and environmental exposures to metals, derived from an
ICP-MS screening program. RJ Audette (Canada)
Biological monitoring of metal exposures in Eastern Europe. M Nordberg (Sweden)
Exposure and toxicity of metal arc welding fumes. PJ Hewitt (UK)
Environmental aspects of arsenic toxicity. TJ Hindmarsh (Canada)
Neurotoxicity caused by lead and methylmercury in children. P Grandjean (Denmark)
Lead toxicity: a paradigm for environmental health sciences research. RA Goyer (USA)
Therapy of Metal Overload and Toxicity
Effects of chelators on cadmium deposits and essential metal levels. V. Eybl (Czech
Republic)
Chelation therapy of lead-exposed children. (to be announced)
The DAMEVAL challenge test as a measure of mercury exposure from dental amalgams. V.
Aposhian (USA)
Paper on therapy of Wilson's disease. (to be announced)
Paper on therapy of Menkes' disease. (to be announced)
Paper on therapy of iron overload. (to be announced)
Epidemiology and Bio-markers of Metal-Induced Cancers
Recent epidemiological evidence on the carcinogeniicity of metals. P. Boffetta (France)
Cancer risk from orthopeadic prostheses. MP Coleman (UK)
Paper on biomarkers of metal exposures and neoplastic transformation (to be announced)
345
VoL 1, No. 4, 1994 Announcements
Members' Registration Fee US$ 350. (for members of the Association of
Clinical Scientists; affiliate members of IUPAC; and members of the Program and
Scientific Advisory Committees who register before 15 january 1995)
Selected Nations Registration Fee US$ 300.(for 50 scientists and physicians from
nations of Eastern Europe, Near East, Africa, South America, Central America, and Peoples
Republic of China who register before 15 January 1995)
Student Registration Fee US$ 300. (for 50 pre-doctoral students, post-doctoral
fellows, and physicians-in-training who register before 15 january 1995, with an
acceptable abstract and a statement of eligibility from their University Dean or
Institutional Program Director). Their doctoral degrees must have been granted after 1
July 1990).
The final deadline for registration is 15 june 1995. Except for
special circumstances, on-site registration will not be available.
Campus Accommodations
The COMTOX Symposium has reserved a large block of single and double rooms for
registrants and accompanying persons in the modern Walter Gage Residence Tower on the
UBC campus. These rooms are comfortably furnished and modestly priced. The 1994 room
rates (in Canadian dollars) are $32 per person per night (plus 15% tax)for
single rooms with shared washroom facilities and $70 per room per night (plus
15% tax) for a room with a double bed (single or double occupancy). The 1995 rates
may be slightly higher.
These prices are for accommodation-only, and do not include meals, which are available at
the Student Union. Accommodation Forms should be sent to the University of British
Columbia Conference Centre, 5961 Student Union Boulevard, Vancouver
B.C., Canada V6T 2C9 (tel: 604-822-1010; fax: 604-822-1001). Requests for
these rooms must be received by 8 June 1995.
Hotel Accommodations
The COMTOX Symposium has reserved a small block of rooms in the sumptuous Landis
Hotel, for registrants and accompanying persons who prefer to stay downtown in
Vancouver and commute to the UBC campus by public bus or taxi. The campus is
approximately 20 minutes by taxi or 30 minutes by bus from the Landis Hotel. The
discounted room rate (in Canadian dollars) will be $125 per night (single or double
occupancy), plus 17% tax. The Landis Hotel's Room Reservation Form should be sent
directly to the Landis Hotel, 1234 Hornby Street, Vancouver, B. C., Canada
V6ZlW2 (tel.: 604-688-1234; fax: 604-689-1762). Requests for these rooms must
be received by 8 June 1995.
Secretariat
F. William Sunderman Jr,. M. D.
Association of Clinical Scientists
Post Office Box 1292
Farmington, Connecticut 06034-1292, USA
Tel: (203) 679-2328
Fax: (203) 679-2154
Registration
346
The regular registration fee and the available discounted fees are as follows:
Regular Registration Fee US$ 450. (registration from 15 jan to 15 June
1995)
Early Registration Fee US$ 400. (registration before 15 january 1995)

				

